# Supplementary material for: Transcriptome immune-regulatory differences between leprosy patients and type 1 reaction patients, before onset of symptoms
Source: PLoS Negl Trop Dis. 2024 Dec 16;18(12):e0011866. doi: 10.1371/journal.pntd.0011866 (PMC11684701; doi:10.1371/journal.pntd.0011866)
Supplement: S1 File — (PDF) [file pntd.0011866.s001.pdf]

Supplementary methods

Parameters used in alignment (STAR) and transcript quantification (Salmon)

STAR was run with the parameters: “sjdbOverhang = 74”, “twopassMode = Basic”, “quantTranscriptomeBan = IndelSoftclipSingleend”, “outFilterScoreMinOverLread = 0.80” and “outFilterMatchNminOverLread = 0.80”. Salmon was run using the parameters “libType = ISR”, “seqBias”, “gcBias” and “discardOrphans”.

Equations for regression models

The corresponding regression models were created in R with the function model.matrix from stats package (v4.0.2) using the factors: sample group, subject ID, stimulation status, age, RIN and sequencing batch. “Sample group” defined libraries as part of the T1R or LEP groups, with the latter as the reference. “Subject ID” indicated which library pairs belong to each recruited individual (a LEP sample was the reference). “Stimulation status” assigned libraries that were stimulated with sonicate or left unstimulated (baseline), with the latter as reference. “Age” was a factor splitting subject into levels “A” if age $\leq$ 24 years and “B” if > 24 years with the cutoff based on the T1R median age. “RIN” was used to create a factor splitting libraries into levels “R1” if RIN $\leq$ 8.2 and “R2” if > 8.2. This cutoff was established empirically to balance the number of libraries between categories. “Sequencing batch” accounted for library pairs in each group that were sequenced in different sequencing runs. Hence a factor with the levels “SB1” and “SB2” was used. The models employed are represented by the following equations:

A) DTE baseline differences:

$$\mathrm{Log_2(CPM)_t} \sim \beta_0 + \sum_{k=1}^{3} \beta_k \cdot \chi_k + \beta_{(\mathrm{T1R})} \cdot \chi_{(\mathrm{T1R})} + \beta_{(\mathrm{stimulation})} \cdot \chi_{(\mathrm{stimulation})} + \varepsilon$$

B) DTE stimulation differences:

$$\mathrm{Log_2(CPM)_t} \sim \beta_0 + \sum_{i=1}^{n-1} \beta_i \cdot \chi_i + \sum_{k=2}^{3} \beta_k \cdot \chi_k + \beta_{(\mathrm{LEP:stimulation})} \cdot \chi_{(\mathrm{LEP:stimulation})} + \beta_{(\mathrm{T1R:stimulation})} \cdot \chi_{(\mathrm{T1R:stimulation})} + \varepsilon$$

C) DTU baseline and stimulation differences:

$$\mathrm{Proportion_t} \sim \beta_0 + \sum_{k=1}^{3} \beta_k \cdot \chi_k + \beta_{(\mathrm{T1R})} \cdot \chi_{(\mathrm{T1R})} + \beta_{(\mathrm{stimulation})} \cdot \chi_{(\mathrm{stimulation})} + \beta_{(\mathrm{T1R:stimulation})} \cdot \chi_{(\mathrm{T1R:stimulation})} + \varepsilon$$

For differential transcript expression, "$\mathrm{Log_2(CPM)}$" represents a transcript's estimated expression (EE) after TMM-normalization and transformed into $\mathrm{Log_2}$ scale with "t" indicating that the same model was fitted for all testable transcripts. Please note that the expression data was not modified. This term is a simplification to represent the use of TMM scaling factors. For the three models, χ is a vector of dummy variables (i.e. "1" and "0") to assign information from libraries to their respective categorical variables β. In model "A", β_0 represents the mean fitted $\mathrm{Log_2(CPM)}$ for LEP at baseline. The term β_k represents the mean expression effect for covariates with k=1,2,3 denoting age, RIN and sequencing batch factors, respectively. $\beta_{(\mathrm{T1R})}$ is the difference between mean fitted $\mathrm{Log_2(CPM)}$ for baseline T1R against the baseline LEP. $\beta_{\mathrm{stimulation}}$ is the mean effect size for *M. leprae* sonicate stimulation across all groups and ε is the residual. In model "A", we are leveraging all libraries (stimulated or not) to boost statistical power to detect baseline differences. In fact, baseline group effects were derived by regressing out $\beta_{\mathrm{stimulation}}$ from all stimulated libraries, then averaging non-stimulated and stimulated libraries for

each group, hence resulting in the coefficients β_0 and $\beta_{(T1R)}$. To guide the contrast of interest, we employed the makeContrasts function from the limma package to create contrast vectors. With model "A" we queried the baseline differences between T1R and LEP by testing the significance of $\beta_{(T1R)}$ adjusting on all other terms.

In model "B", we aimed to boost statistical power to detect group-specific stimulation effects. To that end, we employed a blocking (or paired) regression design by adding the factor with patient IDs as a fixed effect covariate, hence leveraging the fact that each enrolled subject had two measurements (stimulation or not). Each library pair contains valuable information regarding individual-specific biological variance even after stimulation. With this approach, the coefficients for group-specific stimulation effect derived by our model accounted for known covariates (such as age) and any unknown nested ones that could still confound the results of the analysis. In this model, β_0 is the coefficient for a reference LEP subject averaging the stimulated and non-stimulated libraries, after the individual stimulation effect has been regressed out. In contrast to model "A" however, β_0 absorbs all biological noise coming from that reference subject. β_i behaves the same for the i-th individual against the reference sample. β_k (k=2,3) denotes the expression effects for the technical covariates RIN and sequencing batch. $\beta_{LEP:stimulation}$ and $\beta_{T1R:stimulation}$ are the group-specific stimulation effects that have been regressed out from each subject and averaged, thus representing the Log_2FC after accounting for the inter-individual variance. With this model we addressed the stimulation effect for LEP ($\beta_{LEP:stimulation}$) and T1R ($\beta_{T1R:stimulation}$) by contrasting each group's stimulated libraries against their respective baselines and the differential group response by contrasting $\beta_{T1R:stimulation}$ against $\beta_{LEP:stimulation}$ (interaction analysis).

In model “C” proportions indicate the ratio $EE_{(transcript)}/EE_{(gene)}$ for each transcript in which the denominator was the parental gene-level estimated expression. For model “C”, β_0 represents the average baseline expression for the LEP group, β_k has the average effect for the technical covariates and age while $\beta_{(T1R)}$ is the baseline effect size difference for T1R against the LEP. However $\beta_{(stimulation)}$ now represents the mean Log_2FC for contrasting stimulated libraries vs baseline for the LEP group while $\beta_{(T1R:stimulation)}$ holds the effect size for the differential *M. leprae* antigens response between T1R vs LEP. For the differential transcript usage analysis, four contrasts were performed: a) T1R vs LEP baseline differences = $\beta_{(T1R)}$; b) stimulation effect for LEP = $\beta_{(stimulation)}$; b) stimulation effect for T1R = $\beta_{(stimulation)} + \beta_{(T1R:stimulation)}$ and d) differential group response = $\beta_{(T1R:stimulation)}$.
