# Supplementary figures and images for: Transcriptome immune-regulatory differences between leprosy patients and type 1 reaction patients, before onset of symptoms
Source: PLoS Negl Trop Dis. 2024 Dec 16;18(12):e0011866. doi: 10.1371/journal.pntd.0011866 (PMC11684701; doi:10.1371/journal.pntd.0011866)

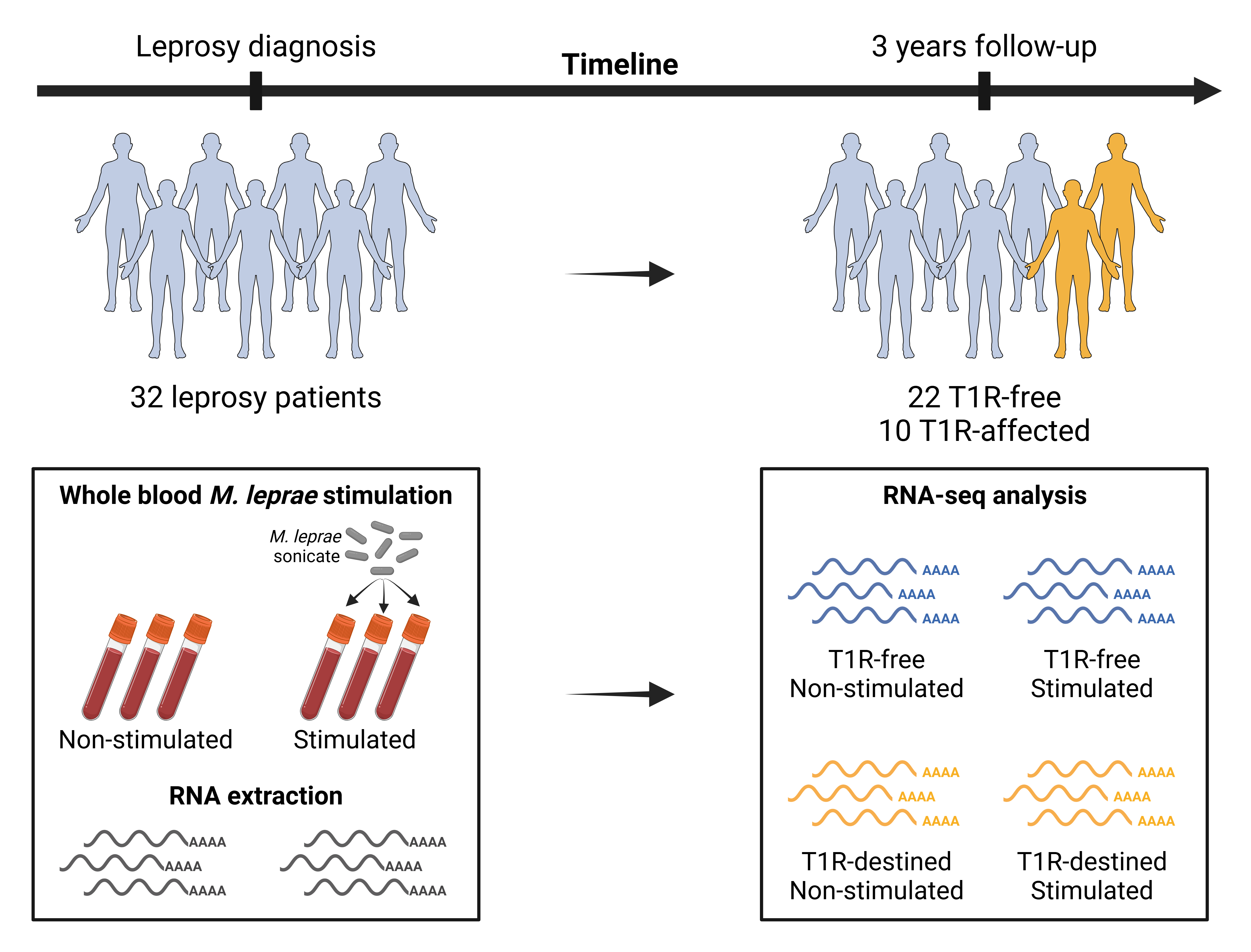

Supplement: S1 Fig — We enrolled 32 patients at the time of leprosy diagnosis and obtained whole blood samples. Aliquots were incubated with media only (baseline, non-stimulated samples) or media + Mycobacterium leprae sonicate (stimulated samples), and total RNA was extracted from all samples and frozen (left box). After three years of follow-up, 22 participants remained T1R-free (LEP group) while 10 developed the T1R reactional state (T1R group). Stored RNA samples obtained at enrolment were sequenced after the three-year follow-up yielding a total of 64 RNA-seq libraries (right box). Created with BioRender.com. (PNG) [file pntd.0011866.s002.png]

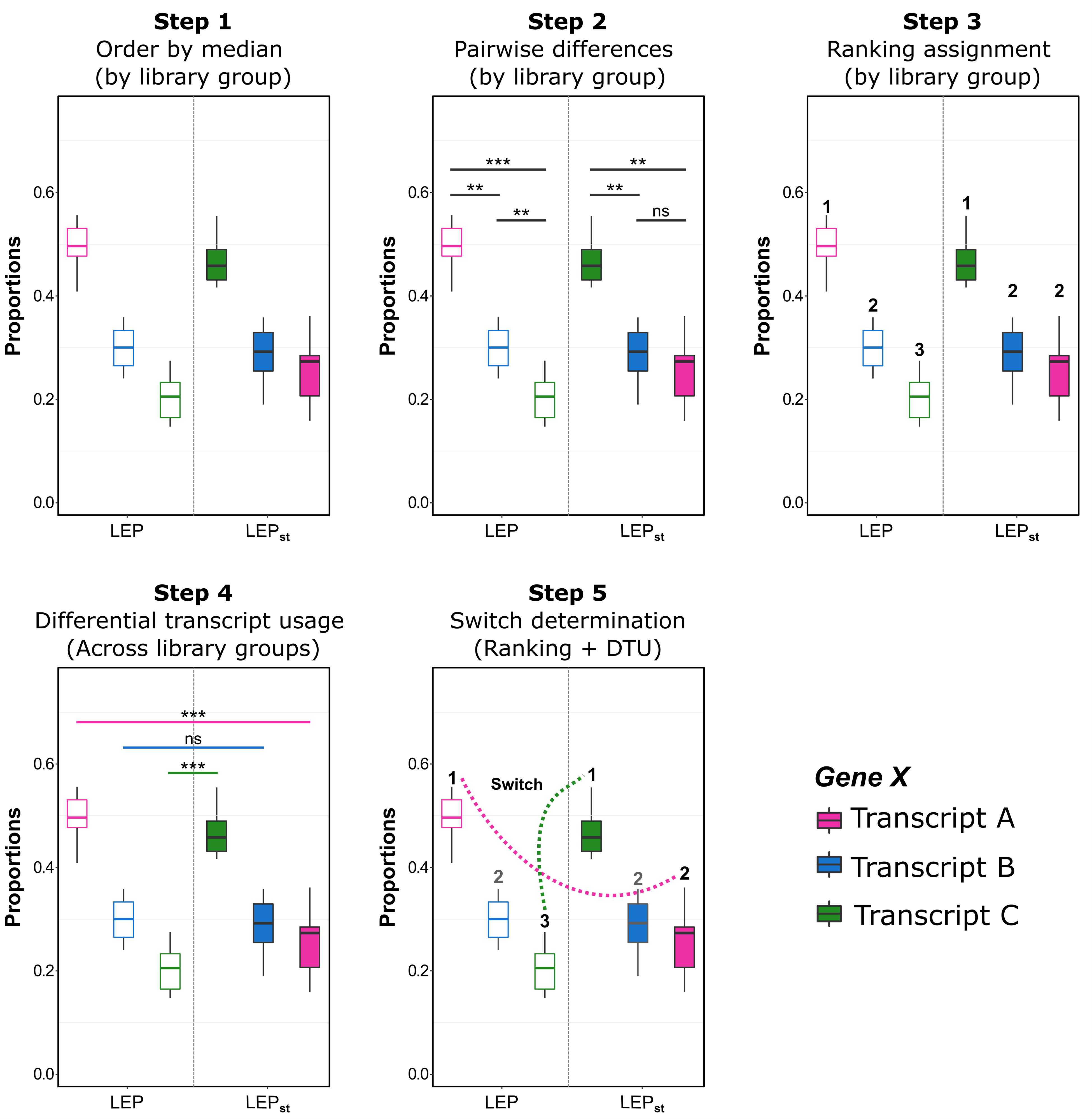

Supplement: S2 Fig — Boxplots in step 1 to 5 represent quartiles for the distribution of fitted usage proportion of each transcript in the corresponding RNA-seq libraries in the context of T1R-free patients. The y-axis indicates fitted proportion values while the x-axis groups the different transcripts by library type (e.g. LEP = T1R-free leprosy patients at baseline condition; LEPst M. leprae antigen-stimulated sample). Step 1: For each of the library groups, we retrieved all transcripts tested for differential transcript usage, for each gene. For each group and condition separately, transcripts were sorted by their medians that were calculated from the distribution of the fitted usage proportions. Step 2: The fitted usage distributions of transcripts were tested via paired sample Wilcoxon tests, within each group and experimental condition. Step 3: Next, we assigned numeric ranks for statistically different usage distributions between two transcripts. If transcripts with different median were not significantly different, they were assigned the same ranking. Step 4: We retrieved the information from differential transcript usage analysis to determine which transcripts were DUT following stimulation with M. leprae antigen. Step 5: Lastly, for all transcripts, we linked the ranking information with their DUT status. If a transcript changed rank following antigen stimulation and was DUT, we called this a switch event. Finally, we checked if a DUT detected at the differential group response had different transcript switches profiles for the LEP or T1R groups following M. leprae antigen stimulation. If so, we also recorded a switch event. (TIF) [file pntd.0011866.s003.tif]

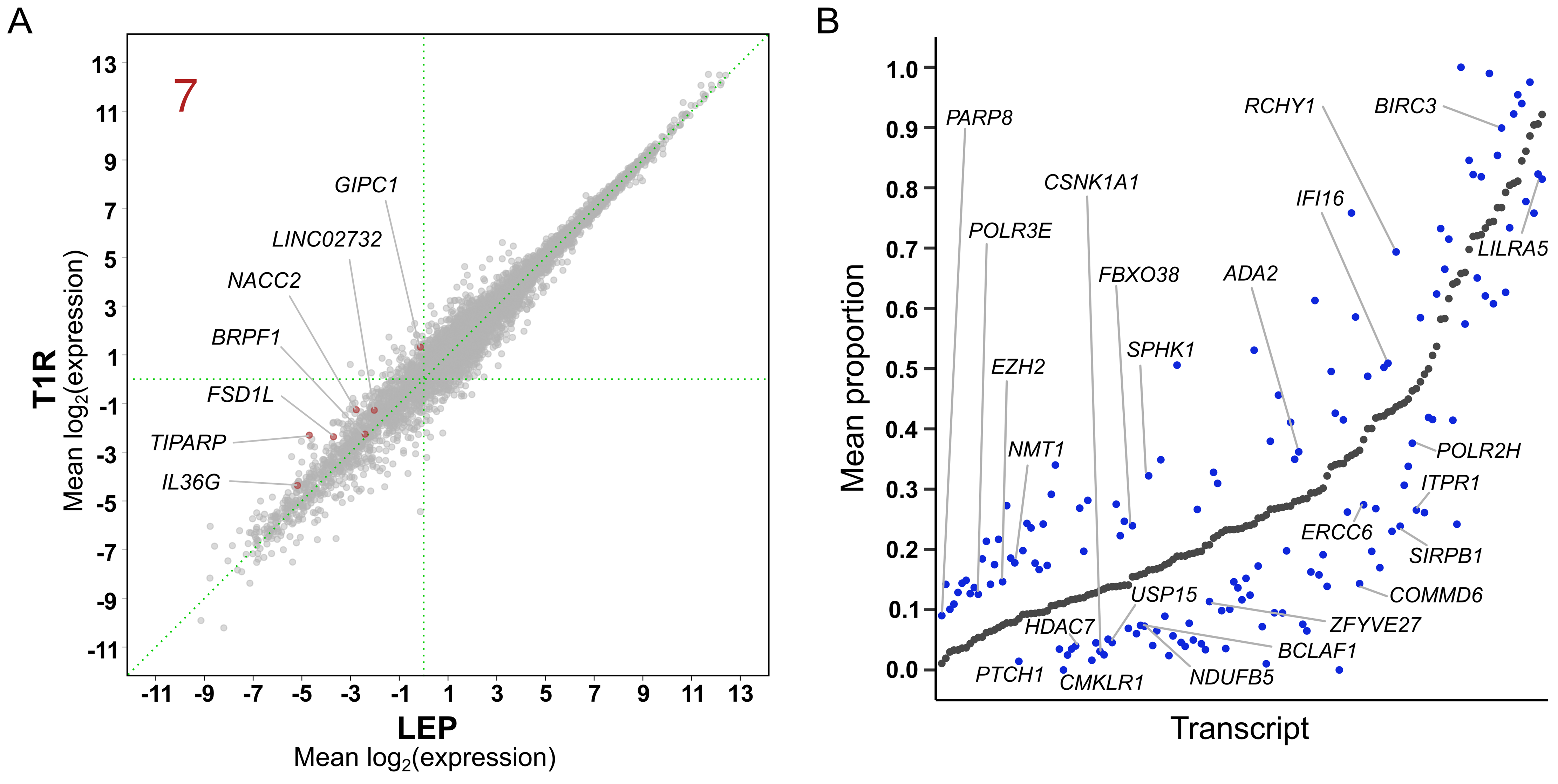

Supplement: S3 Fig — (A) Scatterplot for differential transcript expression at baseline. All tested transcripts are represented as dots and their mean Log2 expression in whole blood is plotted for LEP (x-axis) vs T1R (y-axis) participants. Seven transcripts with mean expression levels that differed significantly between T1R and LEP participants are highlighted as red dots. (B) Transcripts with significant proportion usage difference at baseline. Results are presented as strip plot with the mean fitted proportion usage per group on the y-axis and transcripts ordered by mean proportion usage by the LEP group (shown by grey dots) on the x-axis. Blue colored dots display the mean proportion usage for the corresponding transcripts by the T1R group. (TIF) [file pntd.0011866.s004.tif]

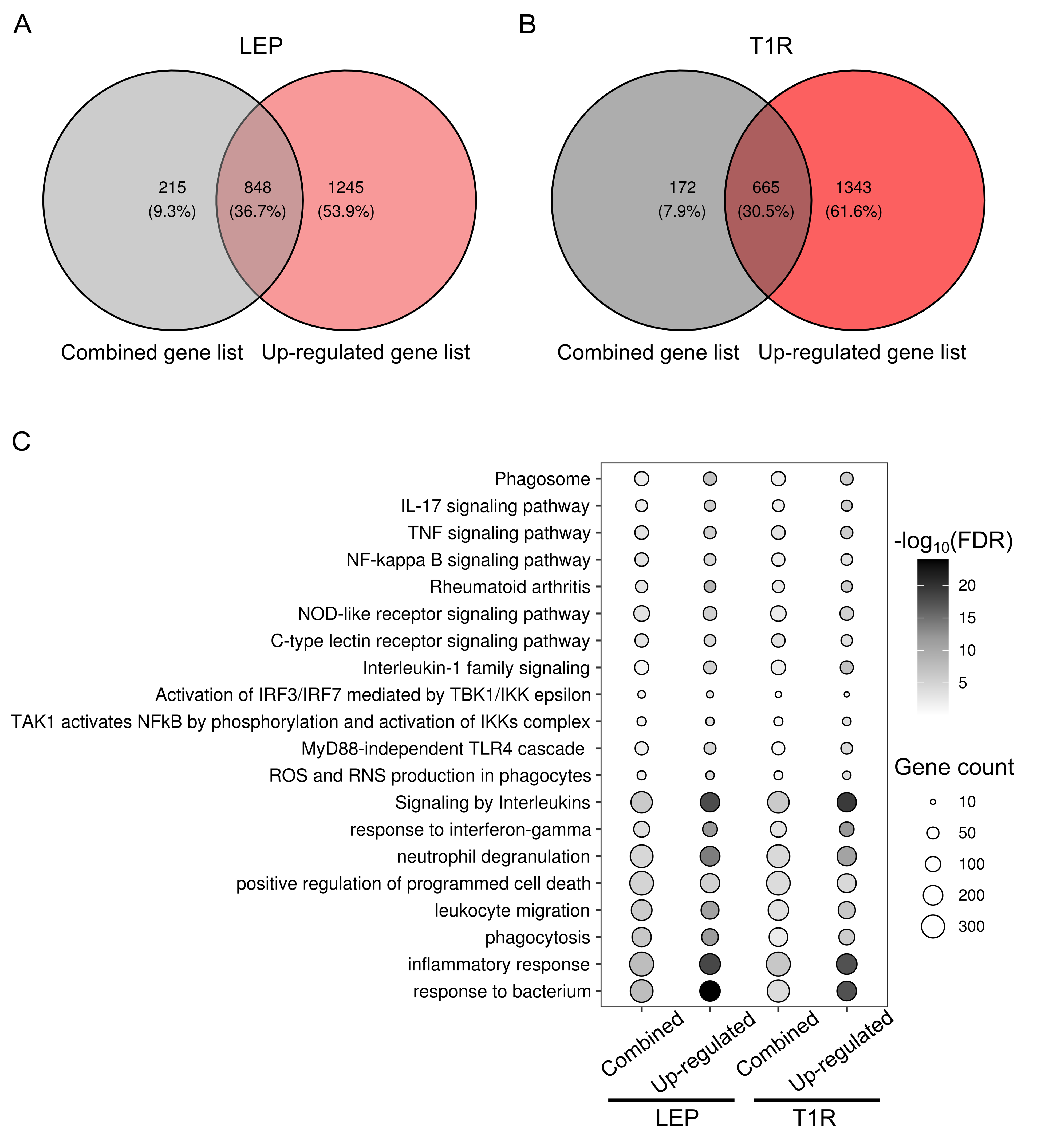

Supplement: S4 Fig — (A) Venn diagram for the overlap of terms obtained by considering only up-regulated or up- and down-regulated (combined) transcripts for the group of LEP patients. (B) Venn diagram for overlap of terms obtained by considering only up-regulated or combined (up- and down-regulated) transcripts for the group of T1R group. (C) Dot plot for a selection of significant GO/pathway terms representing aspects of the immune response to M. leprae antigens. Dot sizes are a function of gene count (implicated by significant transcripts) and shades of gray represent negative Log10 (BH FDR), with darker colors meaning higher significance. (TIF) [file pntd.0011866.s005.tif]

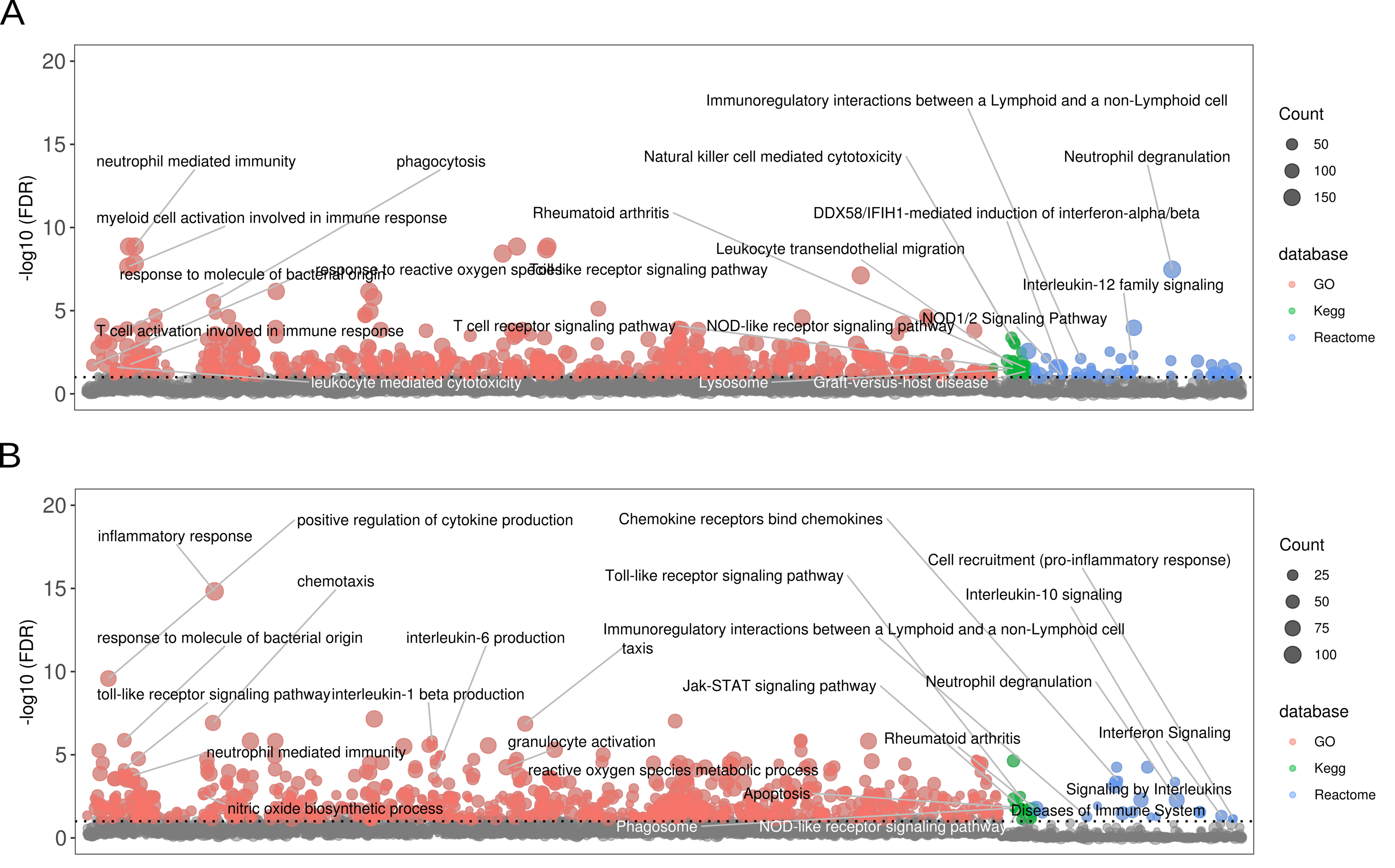

Supplement: S5 Fig — (A) Manhattan plot for GO-terms, KEGG and Reactome pathways with significant enrichment of the 2948 DET with intermediate effect size difference between T1R and LEP responses (0.2 > |Log2FC| < 0.5, Fig 2C, yellow dots). (B) Manhattan plot for GO-terms, KEGG and Reactome pathways with significant enrichment of the 1190 DET with |Log2FC| ≥ 0.5 response difference between the T1R and LEP groups (Fig 2C, green dots). For A and B, the y-axis indicates the negative Log10 for BH adjusted P-values for the terms arranged along the x-axis. The horizontal dashed line represents the 10% FDR cut-off for significant pathways/GO-terms. Dots are sized as a function of the gene number in a term and colors represent the database from which the terms were obtained. (TIF) [file pntd.0011866.s006.tif]

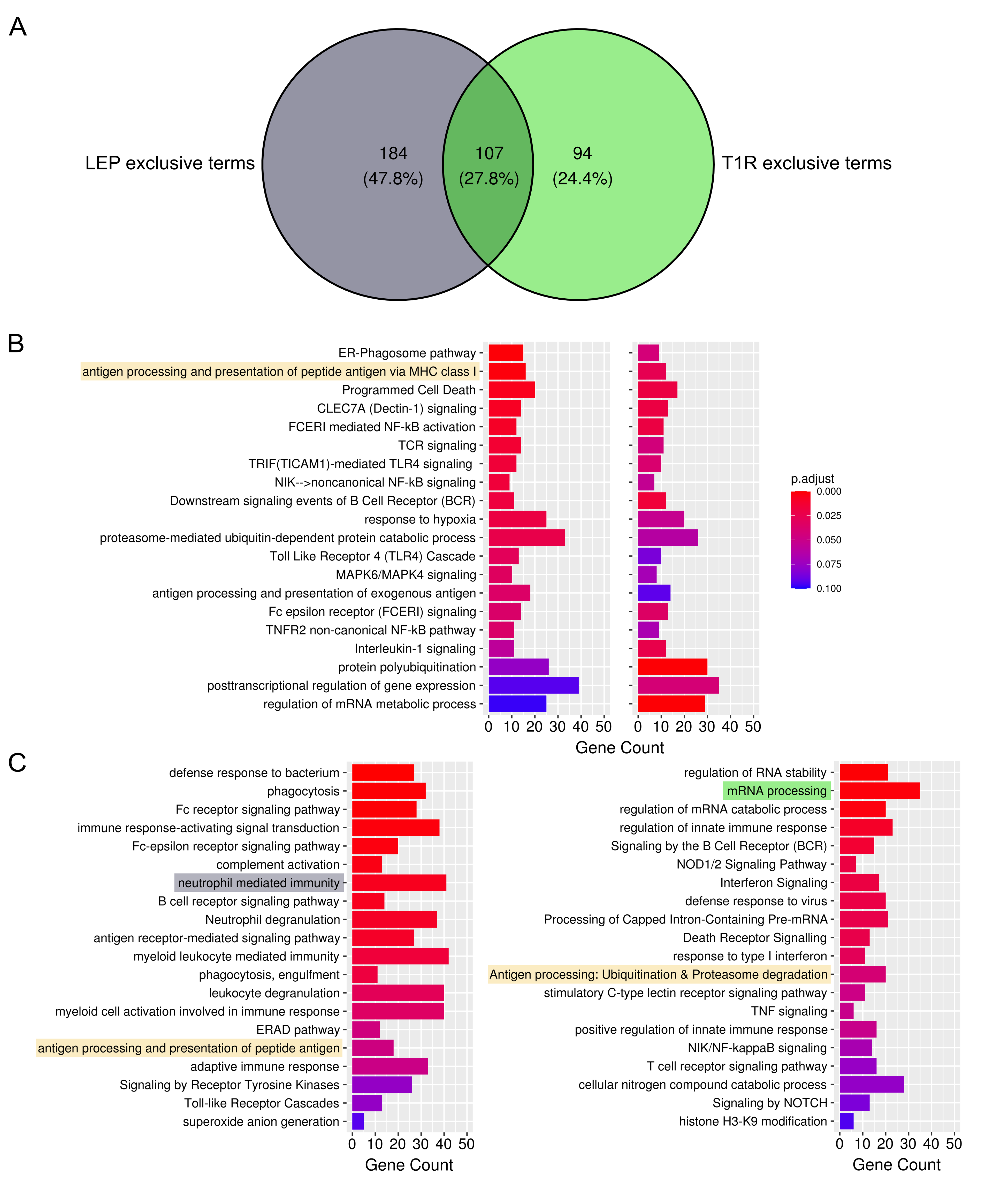

Supplement: S6 Fig — (A) Venn diagram for the number of significantly enriched GO terms and pathways identified by testing lists of genes implicated by positive Log2FC of differentially expressed transcripts (DET) exclusive to T1R (465) or LEP (574). (B) Barplot for a selection of significant terms that represent intracellular defense/immune response among the107 overlapping terms from panel A. The left panel shows results for the LEP group, while the right panel refers to T1R. Terms were arranged along the y-axis as function of increasing Benjamini-Hochberg FDR for the LEP group. The x-axis displays the gene count for each term. (C) Barplots for LEP-specific terms (left panel) and T1R-specific ones (right). Terms sorting was done for each group by increasing Benjamini-Hochberg FDR values. Highlighted in yellow is an example of a physiologically similar term that was found among shared and group specific terms indicating a common biological theme across group-specific responses. The grey-shaded term represents an example of a LEP-specific term which was not enriched in T1R, while green-shaded refers to T1R-specific enrichment. (TIF) [file pntd.0011866.s007.tif]

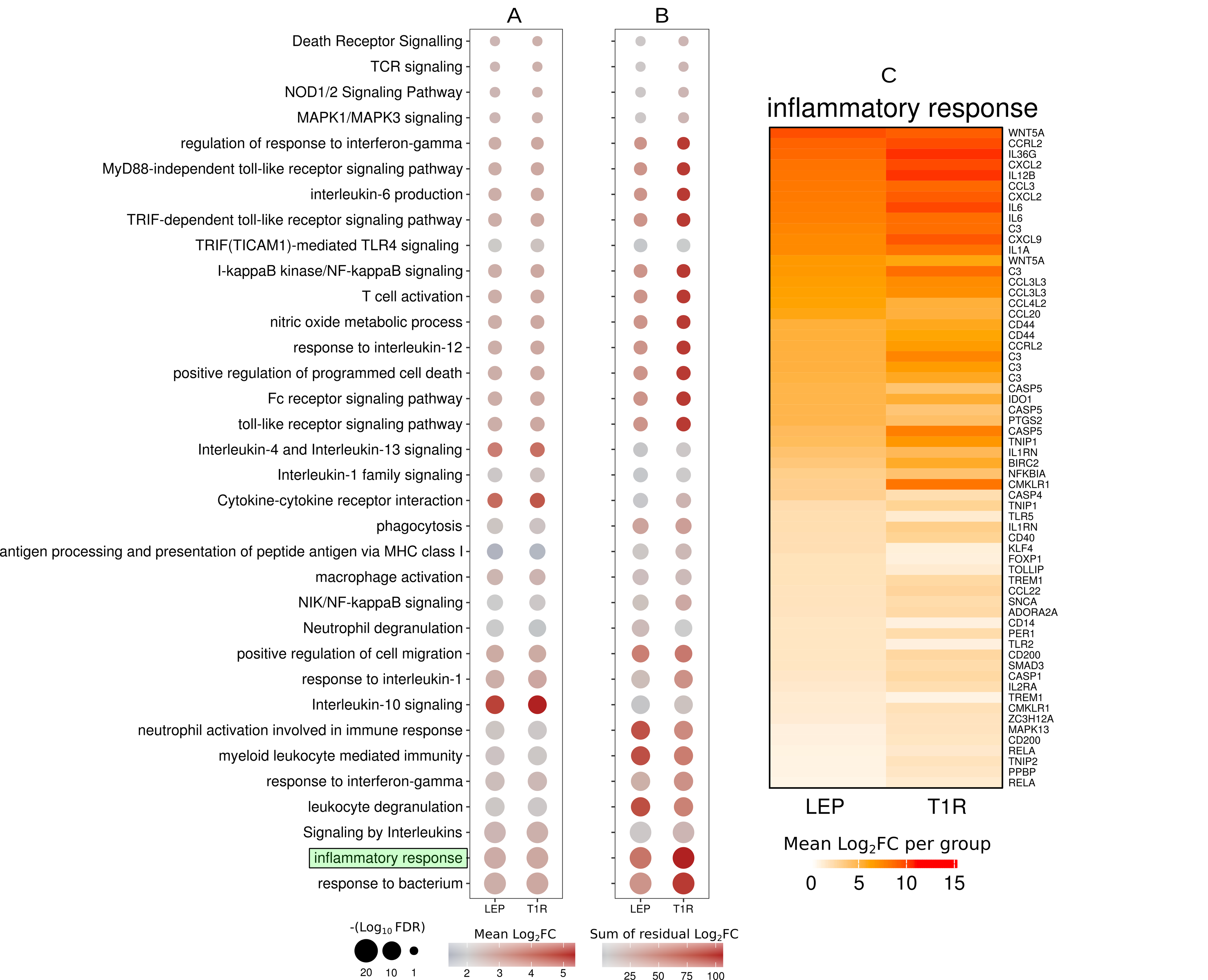

Supplement: S7 Fig — Immune response-linked terms for which the 5639 overlapping up-regulated DET presented in Fig 2D and 2E were significantly enriched. (A) Dotplots of group mean Log2FC of all DET of a given term. (B) Dotplots of the sum of transcript-wise residual Log2FC difference for the LEP and T1R groups. This metric was derived as single value per term and per group as followed: i) Identify the group with the higher Log2FC for a given transcript; ii) the group with the higher value is assigned the difference Log2FC A—Log2FC B while the other group is assigned zero for this transcript. The result for each term is a two-column matrix, each column representing a group, with as many rows as DET in a term enriched; iii) for each group, summing the column of transcript values resulted in one value per term and group. (C) Heatmap for all DET of the GO term “inflammatory response”. The mean Log2FC for all DET was retrieved for each group and ordered by decreasing values of the LEP group. This example shows how the residual Log2FC for enriched terms more strongly displays subtle differences in transcriptional response of the two groups. (TIF) [file pntd.0011866.s008.tif]

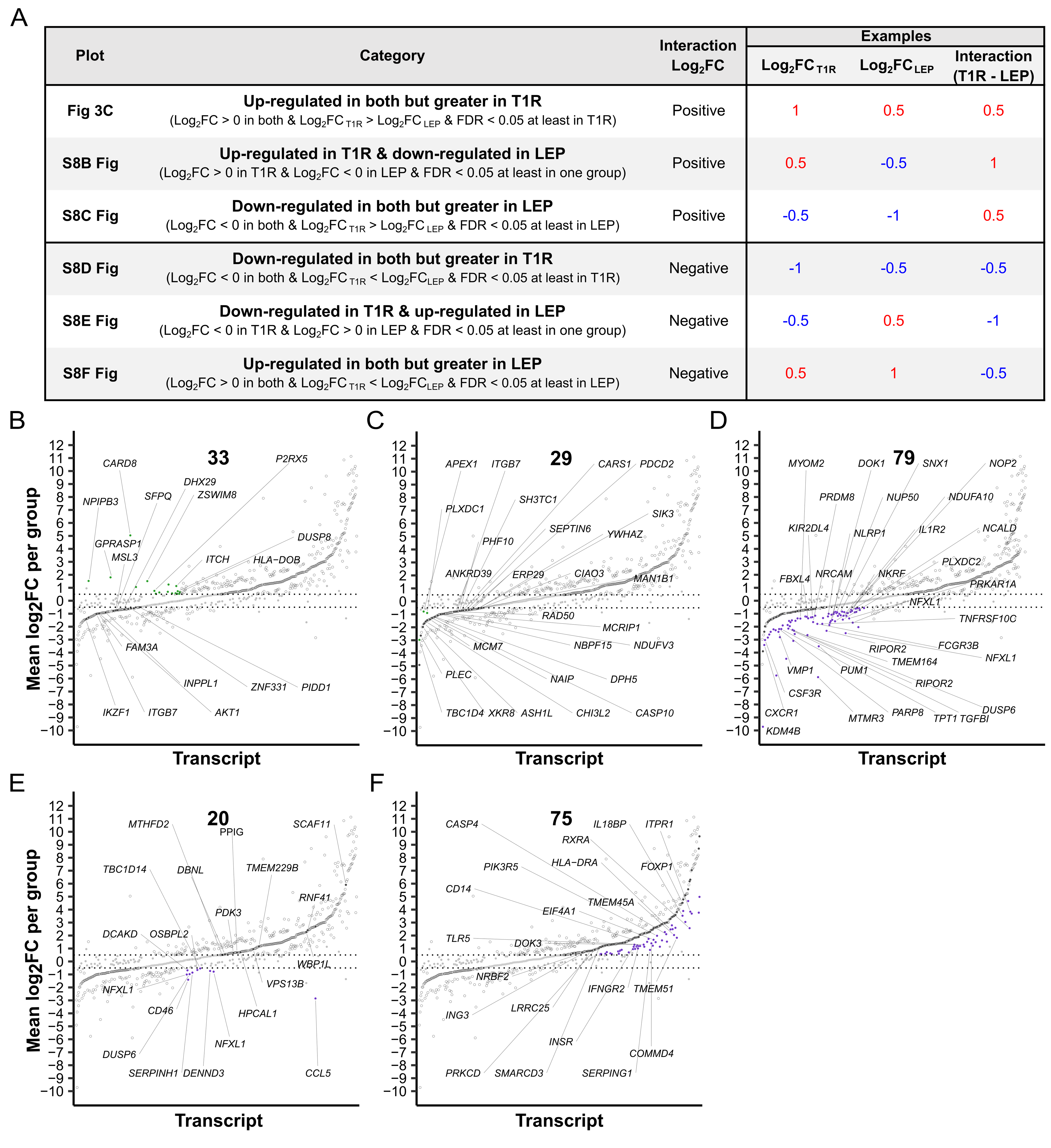

Supplement: S8 Fig — (A) Classes of differentially expressed transcripts (DET) in response to M. leprae sonicate detected via interaction analysis. The effect size difference between two groups is commonly expressed as +/- Log2FC as shown in the examples column. Response differences were grouped into six categories as illustrated here. Transcript details are shown in S3 Table. (B-F) Strip plots presenting the Log2FCT1R and Log2FCLEP from 381 significant differentially expressed transcripts (Fig 3A), separated according to the direction of the changes: B) up-regulated in the T1R and down-regulated in the LEP group (black and green dots), C) down-regulated in both groups but more in the LEP group (black and green dots), D) down-regulated in both groups but more in the T1R group (black and purple dots), E) down-regulated in the T1R group and up-regulated in the LEP group (black and purple dots) and F) up-regulated in both groups but more in the in LEP group (black and purple dots). On the x-axis, transcripts are ordered by their Log2FCLEP. Effect size is represented as mean Log2FC per group (y-axis). Grey dots represent Log2FCLEP. Colored dots display the Log2FCT1R. Open dots are Log2FC for both groups that do not fall in the category presented. The number of transcripts in each category is shown in bold on top of each panel. For panels B, C, and E the majority of corresponding gene symbols are shown for DET. For panels D and F, labeled dots identify transcripts of genes encoding proteins with immunologic/inflammatory functions. (TIF) [file pntd.0011866.s009.tif]

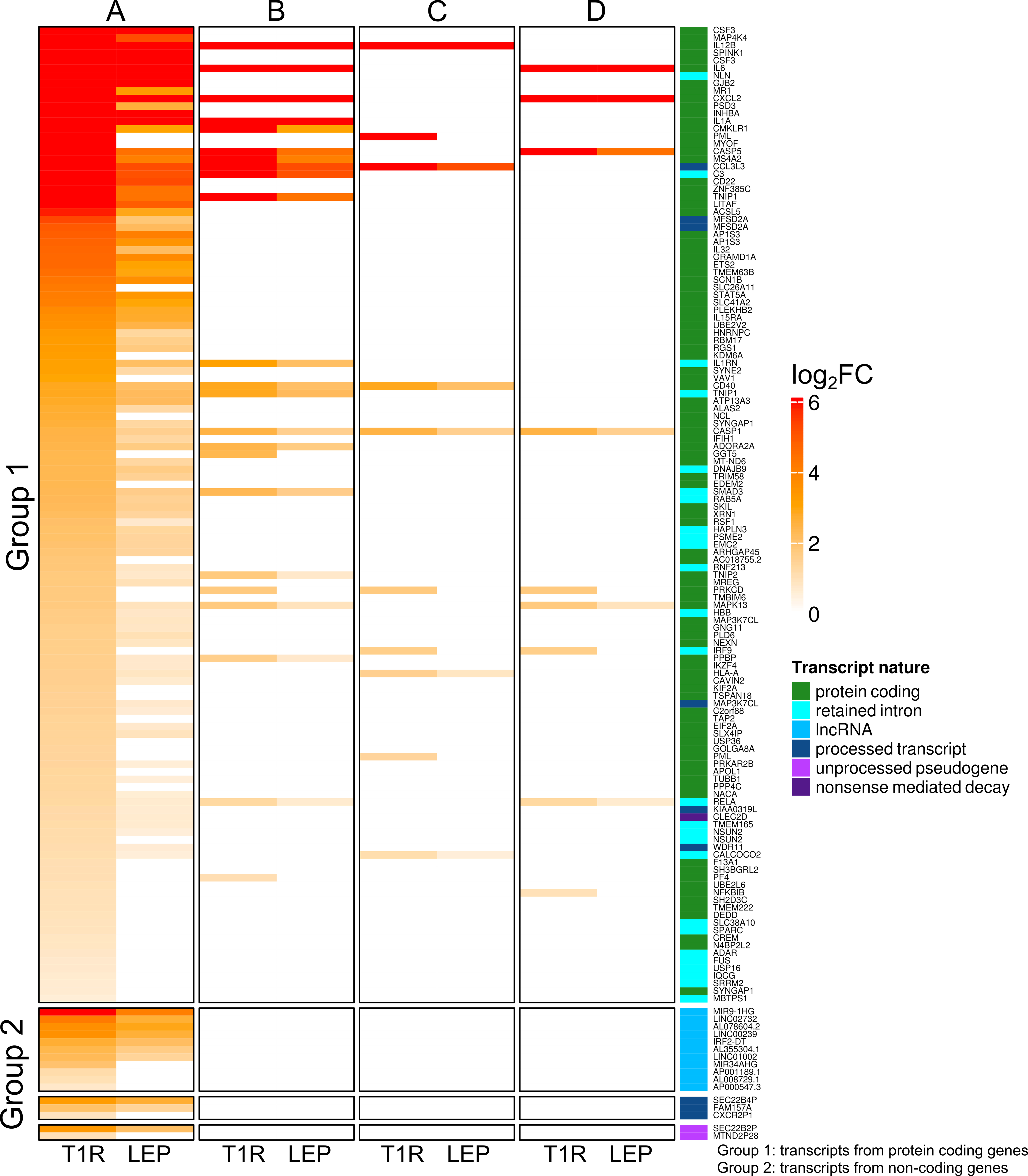

Supplement: S9 Fig — Heatmap for 145 transcripts detected with significant differential response to M. leprae antigens between T1R and LEP patients. Columns present (A) all 145 up-regulated DET with stronger response by T1R, (B) transcripts representing the inflammatory response, (C) transcripts that represent the response to interferon-gamma, and (D) transcripts that represent the NOD-like receptor signaling pathway. The main columns A to D are composed of two sub-columns representing the responses for the T1R or LEP groups. Rows represent the same transcript across all columns and are assigned to group 1 if their parental gene is protein coding or to group 2 if transcripts were annotated to non-coding genes. Colors represent Log2FC values, with strength of responses depicted by scales of red. The right most column present annotations for transcript nature. (TIF) [file pntd.0011866.s010.tif]

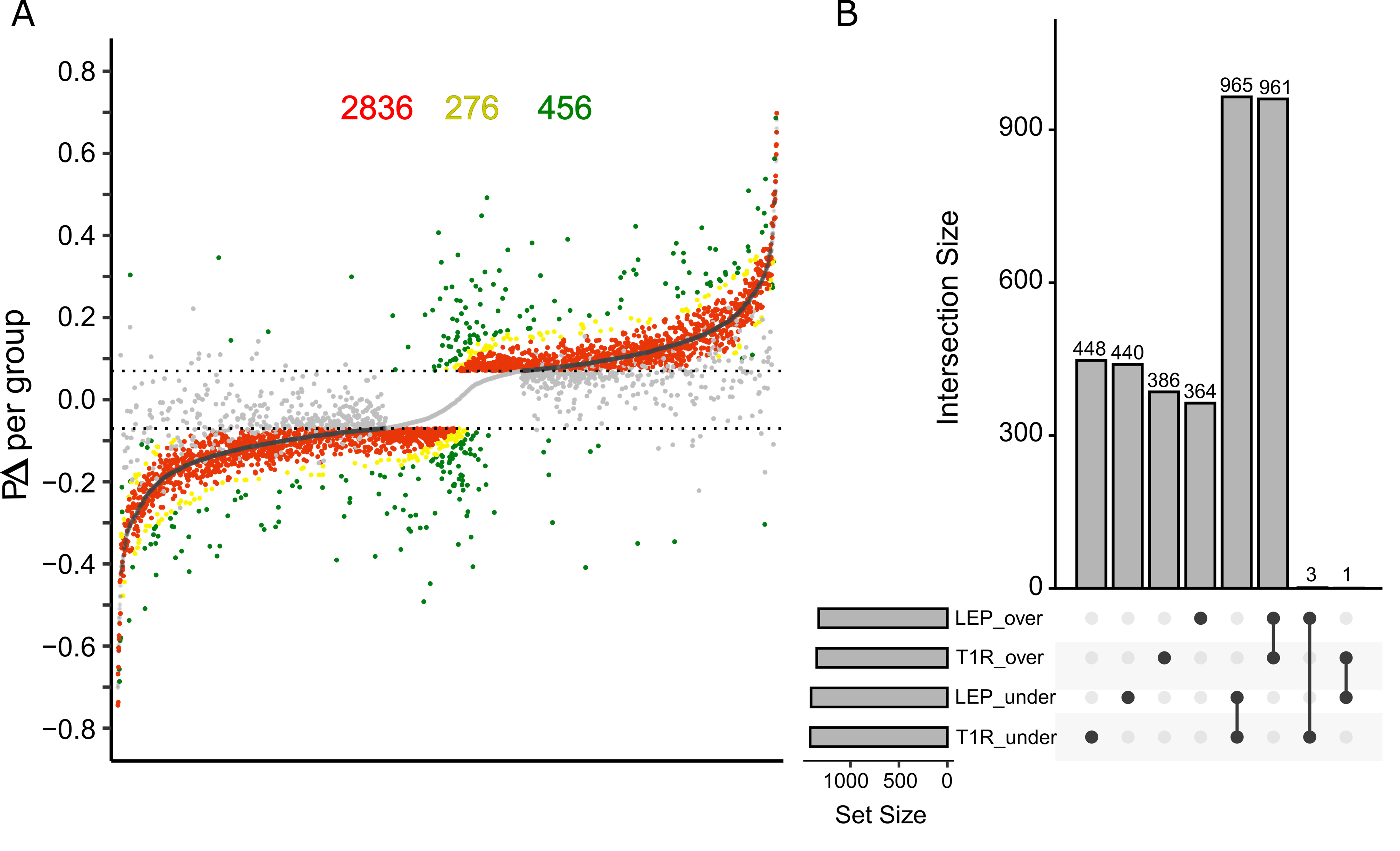

Supplement: S10 Fig — (A) Strip plot presenting the distribution of the mean fitted proportion difference (PΔ) between stimulated and non-stimulated samples by group. Transcripts represented by dots were ordered along the x-axis according to increasing PΔ values for the LEP group. Light gray dots represent transcripts with non-significant PΔ for both groups and horizontal lines mark the | PΔ | ≥ 0.07 threshold. Differentially used transcripts (DUT) for LEP are depicted as black dots and appear as a continuous curve in the center part of the graph while colored dots display PΔ for the T1R group. Red dots show transcripts for which the response difference between groups (interaction) was below the 0.07 cut-off (i.e. |PΔT1R –PΔLEP|< 0.07), yellow represent DUT with 0.07 ≤ |PΔ| < 0.1 and green dots for |PΔ| ≥ 0.1. Colored numbers represent the counts of DUT for the corresponding PΔ intervals. Yellow and green dots are significant interaction DUT, with the green dots indicating the top 30% of the percentile distribution of significant DUT. (B) UpSet plot presenting the differential transcript usage for LEP and T1R responses. The bar chart on the bottom left indicates the total number of increased (over) or decreased (under) transcript usage by the two groups, while the bottom panel identifies how sets intersected. The vertical upper bars indicate the corresponding intersection size (transcript counts). Connected dots indicate DUT that overlap between the categories (sets) shown on the left while unconnected dots highlight DUT detected only for the indicated group. (TIF) [file pntd.0011866.s011.tif]

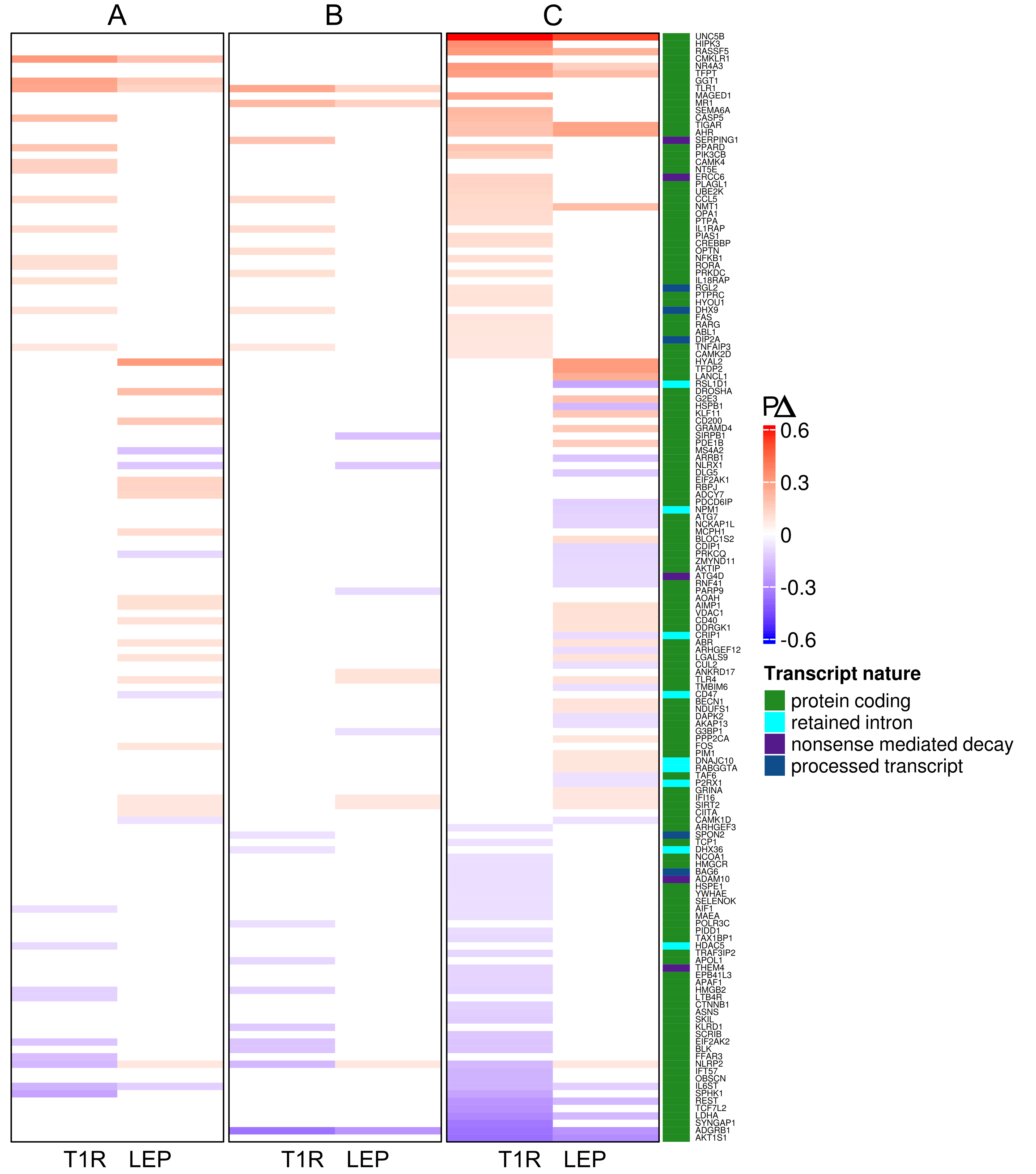

Supplement: S11 Fig — We i) retrieved 1281 DUT that belonged to genes with annotated immunological functions, ii) derived the difference of group responses (i.e. PΔT1R –PΔLEP), iii) sorted the absolute values and selected the top 150 DUT with the largest differences. Next, we selected three GO terms to present how DUT effect sizes compared between groups and how DUT overlapped in the selected terms. For the three main columns, each contains two sub-columns representing effect sizes for the T1R or LEP groups, where PΔ of DUT are listed. Column (A) presents DUT that were annotated to the GO term “inflammatory response”, (B) represents the “innate immune response” GO term and (C) displays DUT for the GO term “apoptotic process”. Each row corresponds to the same DUT across the entire plot since the same gene/transcript may be part of more than one term. Colored cells highlight significant PΔ for a transcript. Non-significant transcripts or ones that were not part of the three selected terms had their PΔ values set to zero (white colored). Shades of red represent positive PΔ values (over-usage in relation to the baseline for each group) and shades of blue represent negative PΔ (under-usage). Transcript biotypes are indicated by a color scheme listed on the right. Gene symbols are shown for each transcript. (TIF) [file pntd.0011866.s012.tif]

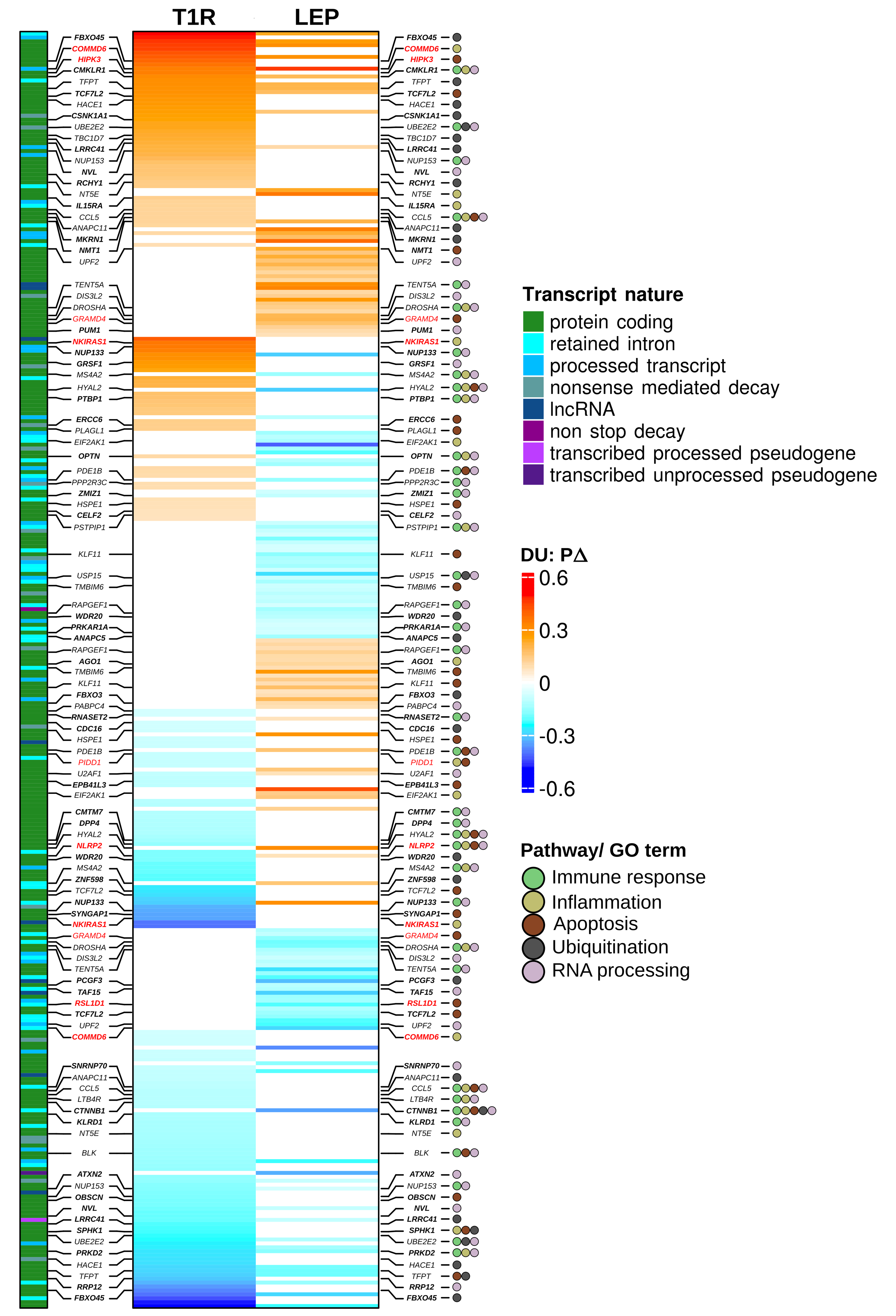

Supplement: S12 Fig — This plot presents the usage response profiles for the 326 differentially used transcripts detected via interaction analysis. The two central columns display the PΔ for the T1R and LEP groups after stimulation with M. leprae sonicate. Shades of red represent positive PΔ values (over-usage) and shades of blue represent negative PΔ (under-usage). Group-specific interaction DUTs (n = 294) responded to the stimulation with differential usage exclusively in one group. They are shown in white for the group where the response was not significant and colored for the group where the response was significant. The remaining interaction DUTs responded to the stimulation in both groups in the same direction but with significantly different size effect (n = 28) or in opposite direction (n = 4), as depicted by the different color shades in the two columns. Rows were sorted by decreasing values of PΔT1R, then re-arranged in three blocks: i) positive PΔ for both groups, ii) opposite PΔ and iii) negative PΔ for both groups. Next, DUT with non-significant changes were assigned PΔ = 0. Given that no gene ontology (GO) or pathway enrichments were found, we searched the annotated function of each implicated gene in the GO, Reactome and KEGG databases. In this process, we detected five major annotation themes: immune response, inflammatory processes, apoptosis, ubiquitination and RNA processing. Transcripts that represented any of these categories are labeled by their gene symbol and the GO/pathway tagged by those genes (colored circles). The left-most column contains annotations for each transcripts’ coding potential or type of non-coding transcript. Gene symbols in red case present examples discussed in the main text, while bold letters indicate transcripts displaying a usage switch. PΔ = difference of transcript proportion between the stimulated vs non-stimulated cells by group. (TIF) [file pntd.0011866.s013.tif]
